# Overview of session

**DOI:** 10.1002/alz70860_105499

**Published:** 2025-12-23

**Authors:** Adesola Ogunniyi

**Affiliations:** ^1^ College of Medicine, University of Ibadan, Ibadan, Oyo, Nigeria; Africa Dementia Consortium, Ibadan, Ibadan, Nigeria; Institute for Advanced Medical Research and Training, College of Medicine, University of Ibadan, Ibadan, Nigeria

## Abstract

Dementia is a growing challenge globally but more so in low and middle income countries where the number of cases are increasing rapidly due to population aging. These countries also have limited potentials for providing adequate care. Some progress have been made in determining the burden of dementia in Africa and many investigators have collaborated in the African Dementia Consortium to provide up‐to‐date data on the epidemiology of dementia, innovative ideas on community engagement to ensure recruitment and retention as well as document genetic risk factors and diversity effects in interpreting genomic data. More recent efforts are concentrated on biobanking and analysis of biomarkers for accurate phenotyping of cases and data comparison with information from the global north. Another area of interest is the determination of appropriate preventive strategies to ensure that the number of cases do not overwhelm the health systems. This perspective session will show‐case new information on epidemiology, community engagement and genomic studies on the platform of AfDC and will show excellent collaboration with the DAWN studies and provide basis for comparison with data from elsewhere and highlight areas for further studies

